# A Systematic Review of Metachromatic Leukodystrophy Newborn Screening: Transitioning from Pilot Studies to the Real World

**DOI:** 10.3390/ijns12040086

**Published:** 2026-10-17

**Authors:** Samuel Waser, Simone Kistler, Laura A. Adang, Paul J. Orchard, Alexandre N. Datta, Neal Sondheimer, Henrik Köhler, Daniel Drozdov

**Affiliations:** 1 Department of Pediatrics, Children’s Hospital Aarau (KSA), 5000 Aarau, Switzerland; 2 University Children’s Hospital Zurich, 8008 Zurich, Switzerland; 3 Faculty of Medicine, University of Basel, 4056 Basel, Switzerland; 4 Division of Neurology, Children’s Hospital of Philadelphia, Philadelphia, PA 19104, USA; 5 Division of Pediatric Blood and Marrow Transplantation and Cellular Therapy, University of Minnesota, Minneapolis, MN 55454, USA; 6 Pediatric Neurology, Developmental Pediatrics and Neurorehabilitation of Northwestern Switzerland, University Children’s Hospital Basel (UKBB), Basel and Children’s Hospital Aarau (KSA), 5000 Aarau, Switzerland; 7 Newborn Screening Ontario, Ottawa, ON K1H 8L1, Canada; 8 Faculty of Medicine, University of Zurich, 8032 Zurich, Switzerland

**Keywords:** metachromatic leukodystrophy, newborn screening, arylsulfatase A, atidarsagene autotemcel, lysosomal storage disorders, systematic review

## Abstract

Metachromatic leukodystrophy (MLD) is a rare lysosomal storage disorder causing progressive neurodegeneration through central and peripheral demyelination, leading to premature death. Recent advances in ex vivo lentiviral gene therapy have developed a potentially curative treatment approach for pre-symptomatic infants with early-onset MLD. This systematic review evaluates the current evidence regarding newborn screening (NBS) for MLD, including screening methodologies, diagnostic pathways, and implementation challenges, using the Swiss healthcare system as an illustrative example. A literature search using keywords MLD and NBS was conducted across five databases on 29 September 2025 and updated on 21 September 2026, along with contacting leading experts of the field up to 1 July 2026, resulting in the inclusion of 27 publications and six conference papers. Multi-tiered screening algorithms combining sulfatide species quantification, second-tier enzyme activity measurement, and genetic confirmation testing can reliably identify MLD-affected infants while reducing false-positive results. Pilot studies and early national implementation programs have confirmed the feasibility of population-based MLD NBS. We consider Switzerland well positioned to integrate MLD into its centralized national NBS program. Several factors, including achieving cost-effectiveness, subtype prediction, and management of uncertain cases, require thorough consideration and represent potentially universal barriers to widespread adoption of MLD NBS beyond pilot programs around the world.

## 1. Introduction

Metachromatic leukodystrophy (MLD, OMIM #250100) is a rare autosomal recessive lysosomal storage disorder caused by a deficiency of the enzyme arylsulfatase A (ARSA) and subsequent accumulation of sulfatides leading to progressive central and peripheral demyelination, neurodegeneration, and, after years of suffering, ultimately death [1,2]. The birth prevalence of MLD in Europe is commonly estimated at around 1 in 100,000 live births, with estimates ranging from approximately 1:54,000 to 1:263,000 live births [3,4].

More than 280 pathogenic mutations in the ARSA gene (OMIM *607574) have been identified to cause MLD. A separate category of ARSA variants is associated with ARSA pseudodeficiency (ARSA-PD), defined by reduced ARSA enzyme activity that does not reach clinically significant levels. Individuals carrying ARSA-PD alleles, including homozygous or compound-heterozygous, show low measured ARSA enzyme activity, but do not develop MLD-associated symptoms [1,5]. Two similar but biochemically different disorders are relevant for newborn screening (NBS) implementation, as they could show as incidental findings: Saposin deficiency, caused by variants in the PSAP gene (OMIM #249900), and multiple sulfatase deficiency (MSD), caused by variants in the SUMF1 gene (OMIM #272200) [1,6]. ARSA activity remains normal in Saposin deficiency but is reduced in MSD, while both conditions show pathological sulfatide elevation. Currently, no disease-modifying treatment options are available for either disorder, and management remains palliative [6].

Although MLD presents with a largely consistent symptom pattern within each subtype, the age of symptom onset and the speed of disease progression vary considerably between patients. The clinical subtypes are distinguished based on the age of disease onset: late infantile form (LI, before 2.5 years), which occurs in approximately 50–60% of cases. Early juvenile disease (EJ) patients become symptomatic between 2.5 and 6 years, while late juvenile (LJ) clinical disease onset is between 6 and 16 years. Juvenile disease accounts for about 20–30% of cases, and an adult form (AD) is associated with disease manifesting after 16 years of age, accounting for approximately 15–20% of cases [3,6].

One of the challenges of MLD is that the early features of disease can be subtle and non-specific. For example, in LI-MLD, many children may be slow to sit independently and crawl, and may fail to fully gain the ability to stably walk independently, which can be difficult to distinguish from a normal toddler gait. Other early features, such as strabismus, may be relatively common and typically benign. Overall, early-onset MLD (LI, EJ) is characterized by subacute loss of motor and cognitive skills over months. The first features of late-onset MLD (LJ, AD) are often related to cognitive and psychiatric symptoms like poor school performance or behavioral change, with motor findings initially mild. Late-onset MLD (LJ, AD) progresses over years, although there is often an acceleration of symptoms around the time of gait difficulties [4,6,7,8]. Because of these challenges to early diagnosis the diagnosis of MLD is often established only after significant loss of function is apparent. Alternatively, the diagnosis can be made pre-symptomatically based on the prior diagnosis of an older sibling, generally having already presented symptoms. The original gene therapy trial used a natural history cohort largely comprising siblings. Generally, to enroll pre-symptomatic patients in the trial, early diagnosis was made due to an older affected sibling, who was not eligible for enrollment.

Apart from biochemical studies, which have evolved considerably in recent years, imaging includes magnetic resonance imaging (MRI) to identify evidence of demyelination in white matter in the central nervous system (CNS). However, imaging tends to lag behind clinical symptoms, and the initial MRI is often normal or may just show slight CNS enhancement, most likely as early symptoms involve peripheral disease rather than the central changes [9]. Characteristic MRI changes in MLD are similar across subtypes and typically present with involvement of the supratentorial white matter with progression to cerebellar matter and atrophy in later stages [9,10,11]. Radial stripes in affected white matter, called a tigroid pattern, are characteristic for leukodystrophy but are not specific to MLD [12]. Visual MRI severity scores have been developed to quantify lesion burden and progression in MLD and were adapted from established approaches in adrenoleukodystrophy; however, these scores are rarely used in practice [9,13,14]. Nerve conduction studies, brainstem auditory evoked responses, and cerebrospinal fluid analyses have also been used in the diagnostic evaluation, and emerging biomarkers such as plasma neurofilament light chain (NfL) have been proposed as additional tools [15,16,17].

Currently, beyond palliative care, only a limited number of therapeutic options are available, each with important considerations. Following the utilization of allogeneic-hematopoietic stem cell transplantation (HSCT) for another inherited leukodystrophy, adrenoleukodystrophy, the field began to explore this option for MLD. This approach relies on identification of a donor, thus introducing challenges with availability of a sufficiently well-matched donor, and post-transplant complications such as rejection and graft-versus-host disease. The proposed mechanism of HSCT in MLD is the replacement of a subset of the brain’s microglia population with donor-derived cells, capable of producing normal physiological levels of ARSA. Unfortunately, this approach has met with limited success in early-onset MLD, where outcomes are generally poor. The failure of traditional allo-HSCT to meaningfully transform MLD is likely polyfactorial, potentially influenced by the rapid disease progression, delay in diagnosis with irreversible neurologic injury, chemotherapy exposure, and the need for supra-physiologic expression. Currently, HSCT is primarily considered for late-onset forms, prior to the onset of major neurologic disability [4].

The development of gene therapy for MLD was able to address many of the barriers to HSCT. Autologous hematopoietic stem cell gene therapy with expression of an intact ARSA gene via ex vivo lentiviral gene transfer, atidarsagene autotemcel (arsa-cel; Libmeldy in Europe, Lenmeldy in the United States), has demonstrated notable benefits when given in early disease stages, particularly prior to the onset of symptomatology in early-onset disease, transforming disease trajectories [6,18,19]. Many children from the original trial achieved normal or near age-appropriate motor and cognitive development [6]. Unfortunately, arsa-cel is currently only approved for a subset of patients with MLD (presymptomatic LI or EJ, and early symptomatic EJ patients). These patients constitute approximately 80% of the MLD population. With the approval of arsa-cel therapy by the European Medicines Agency (EMA) in December 2020, there is also now access to this therapy for children born in many European countries. Quickly, additional regulatory agencies, including the Medicines and Healthcare products Regulatory Agency (MHRA) in the United Kingdom in January 2021, Swissmedic in Switzerland in December 2023, and the FDA in the United States in March 2024, followed with their own approvals [19,20,21].

Newborn screening is the only realistic means of identifying children with MLD who would be eligible for gene therapy, excluding the special case of siblings of an already affected proband. As early treatment has shown substantial benefit, an increasing number of countries are interested in integrating MLD into their newborn screening (NBS) programs to identify affected cases before symptoms develop and the therapeutic window closes. Norway was the first country worldwide to introduce MLD into its national NBS program in June 2024, followed by the addition of MLD to the US Recommended Uniform Screening Panel (RUSP) in December 2025 after the approval of arsa-cel gene therapy, and several US states have since implemented or approved screening [22,23,24,25]. A recent systematic literature review, with summarized evidence up to mid-2025, concluded that a multi-tiered screening algorithm and established confirmatory pathways exist for MLD [26]. Since then, additional prospective pilot data and national implementation decisions have emerged.

The present systematic review updates and extends this evidence by evaluating the performance of multi-tiered NBS for MLD (false-positive rate, sensitivity of detecting affected newborns, and confirmatory pathways), the outcomes of pre-symptomatic treatment, and the real-world feasibility of implementing the full screening pathway. It is based on current international evidence, including screening and confirmatory algorithms, clinical outcomes, cost-effectiveness, ethical considerations, and consensus recommendations, and grades the strength of evidence using the GRADE framework. Given Switzerland’s well-established national NBS program, modern laboratory infrastructure, and access to specialized pediatric care, we will use the Swiss healthcare system as a model for exploration of the feasibility of MLD NBS implementation, and to inform health policy on the potential implementation of MLD newborn screening for Switzerland and beyond.

## 2. Materials and Methods

This systematic review was registered on the International Prospective Register of Systematic Reviews (PROSPERO) on 28 September 2025 (CRD420251156984) and adheres to the Preferred Reporting Items for Systematic Reviews and Meta-Analyses (PRISMA) 2020 statement (Supplementary File S1) [27].

### 2.1. Search Strategy

Relevant research was identified by first searching online databases, including Embase, Medline through Ovid, Pubmed, Scopus, and Web of Science, and secondly by checking the reference list as in backward citation searching. A search string was built from a combination of key words with MLD and NBS with the combinator “AND” and corresponding Medical Subject Heading (MeSH) terms or Emtree terms where possible and customized for each database. To maximize relevant findings, the searches were only limited to works in the English language, human studies, and original research. No search restrictions on publication type, date, country, study design, or report status were imposed. Primary outcomes include studies reporting on MLD NBS, including pilot and national programs, analytical and clinical performance, confirmatory testing, patient outcomes, feasibility, scalability, and cost-effectiveness analyses. The detailed search strings are presented in the Supplementary in Table S1 and the inclusion criteria in Table S2.

A primary database search was conducted on 29 September 2025, and results were organized using Zotero version 6.0.26 [28] and further analyzed using Rayyan Web [29]. During the initial reviewing stage of this manuscript an updated literature search ranging from 30 September 2025 to 21 September 2026 was additionally conducted in order to bridge the time gap between the primary literature search and submission.

### 2.2. Screening and Data Extraction

Titles and abstracts were initially screened by one reviewer and then checked by a second reviewer following the inclusion and exclusion criteria. A full text screening was then performed using the same method, during which additional records were looked for in the reference list. Any mismatches were discussed with an additional third reviewer until a consensus was reached. Reasons for record inclusion or exclusion were documented. Additional records were retrieved by checking the reference lists and by contacting leading experts of the field up to 1 July 2026.

Data was extracted by one primary reviewer and then inspected by a second reviewer using a predefined template, including author, publication year, study design, country of origin, details on multi-tiered screening algorithms documenting which biochemical agents were used, sample sizes, and key findings or main outcomes. After sampling the initial few articles, detailed cut-off numbers, target sample sizes, funding sources, and implementation dates of studies were added. Due to heterogeneity of screening algorithms, cut-off values, and study population, no meta-analysis was conducted, but a narrative synthesis is given instead.

### 2.3. Quality Assessment

Given the heterogeneity of the included studies, a strength of evidence assessment using the GRADE (Grading of Recommendations, Assessment, Development, and Evaluation) framework was used to evaluate outcomes and classified as ‘high’, ‘moderate’, ‘low’, or ‘very low’. A ‘very low’ quality rating suggests that future research is highly likely to alter the existent evidence and suggests that the estimated effect does not match the true effect.

Furthermore, where applicable, risk of bias assessment (Table S3) was conducted using Quality Assessment of Diagnostic Accuracy Studies 2 (QUADAS-2) for diagnostic studies, Newcastle–Ottawa Scale (NOS) for non-randomized and case studies, a measurement tool to assess systematic reviews (AMSTAR 2), and the appraisal of guidelines for research and evaluation (AGREE II) for consensus guidelines.

These were conducted by two reviewers, with any disagreements resolved through discussion with an additional third reviewer.

### 2.4. Ethics

This project does not fall within the scope of the Swiss Human Research Act (Humanforschungsgesetz, HRA) and consequently, ethical approval from the ethics committee (Ethikkommission Nordwest- und Zentralschweiz, EKNZ) was not required for the conduct of this study (BASEC-ID Req-2025-01718).

## 3. Results

A total of 1015 possible records from five databases were identified, from which 248 duplicates were removed. From the 767 references, 84 were identified through title and abstract screening, from which 39 were excluded based on the exclusion criteria. Conference abstracts with corresponding full-text articles were not included. When multiple abstracts were presented on the same study or cohort across different conferences without a published full-text paper, only the most recent abstract was included, leading to the exclusion of 16 conference abstracts. This led to a total of 22 original research articles [6,30,31,32,33,34,35,36,37,38,39,40,41,42,43,44,45,46,47,48,49,50] and six conference abstracts [51,52,53,54,55,56]. One follow-up record of an ongoing study was found as a preprint and added at a later stage [57]. The updated bridge literature search identified an additional 139 records from the five databases, and 29 duplicates were removed. Out of 110 records, 91 were removed by title and abstract screening, leading to 19 articles for full-text reviewing. A total of 15 were removed, leaving 4 additional original research articles [26,58,59,60].

**Figure 1:**
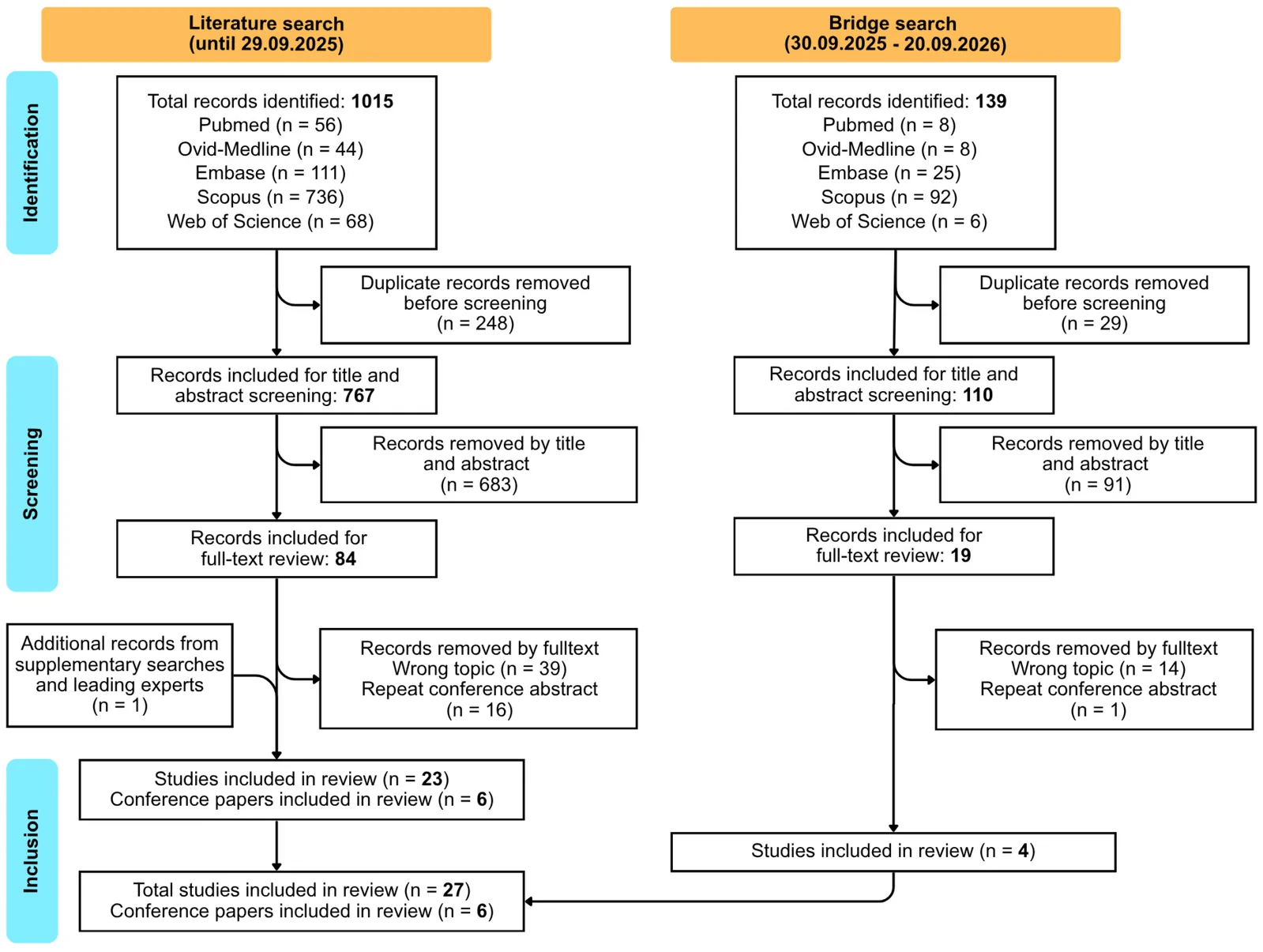
PRISMA flow diagram.

The full summarized details can be seen in the PRISMA flow diagram in Figure 1.

### 3.1. Method Development and Feasibility Studies

Early efforts to establish biochemical and analytical foundations for MLD detection in NBS focused on multiplex immunoassays measuring ARSA protein in dried blood spot (DBS) samples, successfully identifying MLD-affected newborns, including ARSA-PD variants [36]. The studies are summarized in Table 1. Subsequent strategies relying on ARSA enzymatic activity measurement in peripheral blood leukocytes or skin fibroblasts and urine sulfatide quantification proved to be insufficiently specific for population-based NBS, as trace amounts of residual ARSA activity are sufficient to reduce disease severity as seen in ARSA-PD, and leukocyte-based assays are not applicable to DBS samples [6,35,37].

To address these limitations, subsequent studies investigated the direct quantification of multiple sulfatide species in DBS by liquid chromatography-tandem mass spectrometry (LC-MS/MS). Progressive refinement across several retrospective studies identified C16:0-OH- and C16:1-OH-sulfatides as primary first-tier markers for MLD, with additional C18:0-, C18:1-, and C20:0-sulfatides serving as complementary markers [37,46,50,61]. Importantly, these sulfatide profiles were not elevated in ARSA-PD, demonstrating superior specificity over enzyme-activity-based screening alone [50,55]. A multi-center pilot study incorporating data from four NBS centers demonstrated that the combined use of C16:0- and C16:1-OH-sulfatides reduced the first-tier false positive rate to 0.031%, compared with 1.8% for C16:0-sulfatide alone [47]. A more recent study has confirmed C16:0- and C16:1-OH-sulfatides as the most discriminant MLD biomarkers, where in contrast, C16:0- and C18:0-sulfatides were also raised in other neurometabolic disorders [59].

A methodologically significant advance was made when a second-tier assay measuring ARSA activity directly in DBS using a deuterated natural sulfatide substrate and LC-MS/MS was developed, representing the first DBS-based ARSA assay independent of anti-ARSA antibodies and additionally offering a practical confirmatory pathway, as testing could be performed on the original NBS DBS card without requiring a recall for additional blood samples. However, substantial overlap in ARSA activity between ARSA-PD carriers and true MLD cases limits the specificity of ARSA activity measurement as a standalone test, supporting its use as a second- rather than first-tier assay [32].

Population-scale feasibility of the two-tiered approach was subsequently confirmed in a large retrospective study of more than 27,000 de-identified DBS samples, where first-tier screening with C16:0-sulfatide (cut-off ≥0.170 μM) identified 195 samples (0.71%) as screen positive, and second-tier ARSA activity measurement and subsequent third-tier ARSA gene sequencing identified one carrier and one potential MLD case, yielding a two-tier false positive rate of 0.0037% [30]. A related pre-pilot study further recommended lowering the C16:0-sulfatide cut-off to ≥1.65 multiples of the median (MoM) and incorporating C16:1-OH as an additional first-tier marker, after two confirmed MLD cases were found to fall below the initial cut-off of ≥1.8 MoM [49]. Collectively, these studies established the analytical feasibility of a multi-tiered LC-MS/MS-based NBS algorithm using archived DBS samples.

### 3.2. Consensus Statements and Review Articles

Multiple review publications have reached a consensus that NBS for lysosomal storage disorders, including MLD, is analytically feasible but requires a multi-tiered algorithm, combining first-tier sulfatide quantification by LC-MS/MS, second-tier ARSA activity measurement, and third-tier genetic confirmation, to address ARSA-PD in order to achieve adequate specificity and minimize false positive results. Emphasis is also placed on the fact that screening should only be expanded when NBS programs have the capacity for confirmatory testing, treatment and follow-up surveillance [31,35,38,39,40,43,61].

Several expert consensus panels have addressed different aspects of MLD NBS, including standardized assay protocols for international harmonization of laboratory workflows [45], clinical management after a screen-positive NBS test with confirmatory diagnostic pathways, pre-treatment monitoring with clinical-neurological, biochemical, and genetic assessment, and early risk stratification with prediction of MLD subtypes [6,58]. Further factors include immediate referral to a qualified treatment center after a positive screening result, with evaluation within two weeks and biochemical confirmation prioritized over slower molecular testing to preserve treatment eligibility [58]; treatment eligibility and modality recommendations for borderline diagnostic cases [42,58], and structured pre-symptomatic surveillance and long-term disease management protocols [44], including at least 15 years of follow-up after arsa-cel treatment [58]. Across all publications and supporting conference abstracts, early identification through NBS is consistently regarded as clinically meaningful [53,54].

### 3.3. Ongoing Prospective Regional and National Pilot Programs

The largest prospective NBS dataset to date originates from Germany, where a three-tiered program initiated in 2021 screened 109,259 newborns, identifying 381 (0.35%) as first-tier screen positive and confirming MLD in three infants with biallelic pathogenic ARSA variants [26,34]. A recent preprint (full peer-reviewed publication not available) reported updated figures with 359,282 screened newborns as of July 2025, from which nine infants were confirmed to have MLD. Seven received arsa-cel therapy for predicted early-onset MLD (four LI, and three EJ); two with predicted late-onset MLD are being monitored with HSCT planned. The program continues in Germany and has been extended to Austria [57].

In Tuscany, Italy, a two-tier prospective pilot initiated in 2023 has screened 42,262 of a planned 80,000 newborns at the time of reporting, but with no confirmed MLD cases identified in the prospective cohort. A retrospective analysis of 23 known MLD DBS samples demonstrated correct detection in all cases, supporting analytical validity [48].

In Lombardy, Italy, a three-tier prospective pilot study initiated in July 2024 screened 16,130 of a total of 24,598 newborns in the first year, with a target of 100,000. First-tier testing of C16:0-sulfatide and the sum of four C16 sulfatides above the 97.5th percentile led to duplicate retesting with a cut-off above the 99th percentile, which then led to second-tier testing with ARSA activity measurement. Third-tier testing included whole-exome sequencing (WES). No MLD case was identified. Of 58 newborns, in which third-tier testing was conducted, 33 carried ASRA-PD alleles or pathogenic ARSA or SUMF1 variants [60].

A large prospective multi-disorder pilot program in New York, USA (ScreenPlus), initiated in 2021, has screened more than 100,000 infants using a three-tiered MLD algorithm incorporating first-tier C16:0 sulfatide quantification, second-tier ARSA activity measurement, and third-tier ARSA gene sequencing [41].

As of June 2026, several US states, including New York, Pennsylvania, Illinois, Minnesota, Maryland, Tennessee, and Utah, have approved the addition of MLD into their respective NBS programs. Implementation occurred in Pennsylvania as the first state, followed by Illinois, while New York has transitioned to statewide screening by a pilot program [24,26,39,62,63,64]. As MLD was added to the federal US Recommended Uniform Screening Panel, we can anticipate MLD being added expediently to statewide screening programs [22,24].

Pilot studies have been initiated in France, including an assay validation study at Hospices Civils de Lyon and a prospective pilot at Rouen University Hospital, as well as in Saudi Arabia at King Fahad Medical City Hospital; however, no data have been published to date [26,47].

The prospective studies and national implementations are presented in Table 2. Effect measures (e.g., FPR) are reported as extracted.

### 3.4. Cost-Effectiveness Analysis

The first and only cost-effectiveness study of MLD NBS was published in the UK in 2024 and estimated, using a decision-tree framework and assuming an incidence of 1:100,000 live births, that NBS would identify about 7.04 MLD cases per year eligible for treatment compared with 2.60 eligible cases without screening, leading to an incremental cost-effectiveness ratio (ICER) of £33,212 per quality-adjusted life-year (QALY) gained. The additional expenditure was primarily driven by arsa-cel treatment costs for the four additional patients. This was below the willingness-to-pay threshold of £50,000 per QALY used for highly severe conditions, suggesting that MLD NBS may be cost-effective in the UK setting, but would increase total health-system costs [33]. Two conference abstracts from the same authors reported comparable findings, where one investigation concluded that NBS in the United States would be a cost-effective use of resources [51]. The second abstract estimated that NBS in California would avert 5.09 premature deaths, leading to a benefit-to-cost ratio of 2.9 and a net monetary benefit of $70 million and an estimated societal cost of $34 million if screening would not be performed [52]. Overall, all three analyses suggest meaningful health benefits and economically favorable outcomes, supporting the inclusion of MLD into routine NBS programs.

### 3.5. Strength of Evidence and Risk of Bias

According to the GRADE assessment, the quality of evidence of the NBS test performance was rated moderate, suggesting that multi-tiered algorithms can achieve a low false-positive rate when optimized (Table S4). Sensitivity for detecting affected newborns (false negative rate), confirmatory pathways (second and third tiers), and patient outcomes of early treatment were graded with low or moderate quality, while patient outcomes of the full multi-tiered screening pathway and treatment were rated very low quality, due to small case numbers and lack of follow-up. Common downgrading factors include heterogeneity, imprecision, and publication bias, particularly possible industry involvement.

Risk of bias was not assessed in conference abstracts [51,52,53,54,55,56], narrative reviews [31,35,38,39,40,43], economic studies [33], or when no NBS MLD results were described [41,45], leaving 18 studies, which were formally assessable. Method development and feasibility studies [32,36,37,46,50,59] showed some to high risk of bias due to possible spectrum bias. Larger pilot programs assessed with QUADAS-2 [30,47,48,49,60] showed low to some risk of bias due to transparent reporting and consecutive NBS cohorts, while other cohorts assessed with NOS [34,42,57] showed low to moderate risk of bias due to absent comparators and preprint status. One systematic review [26] showed moderate confidence and two consensus guidelines [6,44,58] showed some risk of bias due to industry affiliation of co-authors.

## 4. Discussion

The approval of a transformative gene therapy for early-onset MLD has led to a novel challenge: a therapy without identified patients [65]. It is clear that NBS for MLD is now feasible for population-wide implementation, when reliable DBS-based screening algorithms, established confirmatory pathways, and structured clinical management of screen-positive infants are in place [26]. Since the discovery of the benefit of early dietary treatment for phenylketonuria (PKU) by Horst Bickel in 1953 and the subsequent development of the Guthrie test by Robert Guthrie in 1961, NBS has become one of the most successful measures of secondary disease prevention [66,67]. Screening newborns for MLD can drive a similar success by transforming the course of a fatal disease using a one-time preventative therapy.

MLD now meets established Wilson and Jungner screening criteria, given its disease severity, detectable presymptomatic phase, reliable screening algorithms, and the significant improvement of disease outcomes when treated pre-symptomatically in early-onset MLD [68,69]. In our analysis we found that early identification of MLD through NBS has substantial public-health value, and expansion of national NBS programs globally is feasible and strongly recommended.

Some important considerations remain prior to widespread MLD NBS implementation. First is the importance of multi-tier testing to reduce false positives. The standard recommendation is first-tier LC-MS/MS-based quantification of sulfatide species (C16:0 and C16:1-OH). As more regions implement MLD NBS, there has been the expected refinement of reporting thresholds. For example, the UK pre-pilot study showed the importance of cut-off adjustments, as the original C16:0 threshold risked missing MLD-affected infants, leading to the recommendation of a lower C16:0-sulfatide cut-off and incorporation of C16:1-OH-sulfatide [49]. Subsequently, a multi-center analysis confirmed that C16:1-OH-sulfatide determination improves first-tier testing precision [34,47,57,59]. Second-tier recommended testing is ARSA activity measurement. Finally, genetic third-tier testing with confirmatory follow-up is strongly recommended. This approach substantially reduces false-positive results and helps to distinguish true MLD from ARSA-PD, MSD, and Saposin deficiency. The German, Austrian, NY, Tuscany, and Lombardy pilots support the feasibility of broad implementation without false positive identification [34,41,48,57,60]. Additionally, the Lombardy pilot illustrates the sensible nature of cut-off determination: the 1st tier threshold at the 97.5th percentile required duplicate retesting of 6.41% of the original DBS cards, where 1.53% (58 newborns) remained above the cut-off and proceeded to ARSA activity measurement. Genetic 3rd tier testing then prevented the recall of 58 newborns with low ARSA activity, most of which carried ARSA-PD alleles [60].

In addition to the technical components of NBS implementation, there must also be a clearly defined clinical pathway. Identified patients should be referred urgently for evaluation by an expert metabolic and neuropediatric team. The expert referral team will support subtype prediction, as identification of predicted phenotypes guides treatment decisions and gene therapy eligibility. The European consensus recommendations outline the steps for subtype prediction, which largely relies on genotype and family history [6]. Novel genotype combinations and variants of uncertain significance (VUS) will complicate treatment decisions, although the international MLD community has aligned with monitoring recommendations in these cases [6,44]. MetabERN (European Reference Network for Hereditary Metabolic Disorders) has established an expert advisory panel to support decision-making in ambiguous cases, particularly when genotype-phenotype correlations are uncertain [6,42]. Immediate referral to a qualified treatment center within two weeks of a positive NBS result is strongly recommended, prioritizing biochemical confirmation, as prolonged genetic work-up may cost treatment eligibility. In countries or healthcare systems without access to centers with fitting diagnostic equipment or gene therapy, early cross-country referral to qualified treatment centers may be necessary [58]. The presence of harmonized peer-reviewed recommendations and availability of experts for guidance underscores the readiness of MLD for NBS implementation, and it is anticipated that as more experience is gained with unclear genotypes, greater accuracy will be obtained.

Ideally, countries would pair approval of the therapy with implementation of screening. Norway was the first country to implement MLD into its NBS program in June 2024, and it marks a pivotal milestone for other high-income countries considering panel expansion [25]. In the United States, MLD was added to RUSP in December 2025, following the approval of gene therapy [22,23,24]. In contrast, while therapy is available, the UK National Screening Committee decided in November 2025 against adding MLD into the NBS panel, due to unclear cut-off values, gaps in test accuracy, and uncertain cost-effectiveness [70,71]. Under immense public pressure, Prime Minister Keir Starmer promised to reconsider this decision [72]. Also the new Prime Minister Andy Burnham “will make sure we look at this again”.

Generally, the requirements for MLD implementation are, firstly, the existence of an already established and integrated NBS program. Secondly, the biochemical testing capacity to run multi-tiered algorithms using standardized assays, including genetic and confirmatory testing. Thirdly, when infants are deemed treatment-eligible, timely access to treatment along with structured monitoring and management must be ensured. Fourthly, on a governmental level, overcoming the legal and regulatory barriers to adding a condition to the panel, as well as securing funding for the screening costs and reimbursement of a high-cost therapy. Lastly, access to a psychosocial support framework, since a diagnosis, and even more so, ambiguous cases or false-positive results, can lead to a significant psychosocial burden for families.

As a country with early approval of gene therapy and a robust NBS system, Switzerland is well positioned to further develop NBS for MLD. Switzerland-wide NBS was introduced in Zurich in 1965 and achieved nationwide coverage by 1975, while the number of target disorders has gradually increased to eleven: PKU, cystic fibrosis, congenital hypothyroidism, glutaric aciduria type 1, medium-chain acyl-CoA dehydrogenase deficiency (MCAD), maple syrup urine disease, galactosaemia, congenital adrenal hyperplasia, biotinidase deficiency, severe combined immunodeficiency (SCID), and, since 2024, spinal muscular atrophy (SMA) as the most recent addition [73,74,75]. Screening is performed using DBS collected within the first 72 to 96 h after birth and analyzed centrally at the only NBS laboratory located at the University Children’s Hospital Zurich, with established referral structures for metabolic and genetic disorders. Testing capacity and infrastructure are largely already in place: the laboratory operates fully automated LC-MS/MS platforms with validated DBS extraction workflows that could support first-tier sulfatide quantification, and genetic confirmation testing is already performed for several disorders [60,76,77,78]. The international proposed ARSA enzyme activity testing, however, is not directly transferable, as enzyme assays in Switzerland currently run on LC-MS/MS, and existing enzyme assays instead operate on fluorometric platforms [75]. Notably, as proposed by the Lombardy pilot study, once a standardized 1st tier sulfatide quantification assay is available (C16:1-OH), WES could be implemented directly as a 2nd tier test, bypassing the technically demanding enzymatic analysis, ultimately circumventing this limitation [60]. Nonetheless, this centralized structure permits uniform implementation, quality control, and national outcome monitoring.

As a rare disease, the expected number of MLD cases in Switzerland would be low, but not negligible. Based on 78,153 live births in Switzerland in 2025, approximately 0.38 to 1.85 MLD cases per year would be expected using the European prevalence range of 1:54,000 to 1:263,000 live births [3,79]. These low numbers support a national rather than a regional implementation and underline the importance of longitudinal data collection. Furthermore, deaths from MLD would be avoidable as the treatment was regionally approved in 2023 [20].

Adding MLD to the Swiss NBS panel requires federal authorization from the Bundesamt für Gesundheit (BAG) under the Federal Act of Human Genetic Testing (GUMG) with advisory review by the Federal Comission for Genetic Testing (GUMEK). This process is further constrained by the Act’s proxy-consent framework, as genetic mass screening may only be performed with informed consent, which parents or guardians provide on the newborn’s behalf [80]. The process is feasible, as demonstrated in SCID: introduced in January 2019 under a five-year, time-limited authorization, it was converted to a permanent authorization by the BAG following re-evaluation, showing that a potential initial MLD pilot screening would likewise be achievable [75]. A Swiss cost-effectiveness analysis is preferable before implementation, as MLD is an ultra-rare disease, and arsa-cel is among the highest-priced therapies worldwide with reported acquisition costs of Lenmeldy of $4.25 million in the United States for a one-time treatment, making it one of the most expensive approved medical substances globally [81]. The UK cost-effective analysis provides an important reference point; however, the UK model cannot be directly transferred to Switzerland, as treatment prices, healthcare costs, reimbursement rules, discount rates, and willingness-to-pay thresholds differ. A Swiss model should therefore incorporate several perspectives, including screening and confirmatory testing costs, treatment costs, long-term care costs, and family burden with productivity losses. Additionally, the pre-treatment evaluation and recommended mandatory post-treatment surveillance of at least 15 years as advised by the European multidisciplinary expert panel, are very resource-intensive and will generate substantial downstream costs, which may not be fully reimbursed and should be included in such a model [58]. Given the rarity of MLD, even small changes in incidence assumptions, treatment eligibility, or therapy pricing may substantially influence the cost-effectiveness evaluation.

The potential benefits of integrating MLD into NBS programs are substantial, as irreversible neurological damage in treatment-eligible infants may be avoided, rapid access to treatment may be made possible, and in non-eligible infants, whether due to predicted late-onset disease or where the disease course is already too advanced, proper surveillance and symptomatic treatment along with early family counseling may be possible. As NBS is implemented, it is important to leverage a testing strategy that reduces false-positive results, as this could lead to a significant psychosocial burden for families, and the identification of infants with uncertain phenotype prediction and difficult treatment decisions [26]. As such, structured family counseling, psychosocial support, and transparent decision-making should also be provided.

Finally, our GRADE assessment supports a careful but favorable certainty of the gathered evidence. Screening test performance was rated as moderate quality, reflecting consistent evidence that optimized multi-tiered screening algorithms can achieve low false-positive rates. Evidence for sensitivity, confirmatory pathways, early treatment outcomes, and full screening-to-treatment pathway was rated very low to moderate, largely because of small numbers of true MLD cases, heterogeneity in assays and cut-offs, limited follow-up, and possible industry affiliations in published evidence. Notably, these limitations do not negate the rationale for screening but argue for implementation within a well-structured evaluation and decision framework, where each screening program should evaluate MLD integration by cost-effectiveness models, regional or national technical feasibility, availability of expert councils, and careful benefit-harm considerations.

### Future Directions

Several important gaps remain to be addressed to advance MLD NBS. Firstly, long-term outcome data is urgently needed, as the number of NBS-identified infants with adequate follow-up remains very small, multi-center international registries will be essential, and unifying an approach to monitoring will prove important. Secondly, subtype prediction must be improved to address the limitations of current phenotype–genotype correlation, as subsequent therapy decisions depend on distinguishing between early- and late-onset forms. Thirdly, standardization of screening algorithms across laboratories and countries remains a priority, with particular attention to cut-off values, with the goal of reducing the retesting burden. Finally, for Switzerland specifically, a cost-effectiveness analysis, evaluation of available structured psychosocial support frameworks, and possible pilot program should be evaluated before full implementation.

## 5. Conclusions

In conclusion, the currently available evidence supports the consideration of MLD for inclusion into the Swiss NBS panel. International feasibility, prospective, and pilot studies, and especially national implementation in Norway and the United States, demonstrate that a DBS-based multi-tiered screening approach is technically feasible and suitable for integration into already established NBS workflows on a population-wide level. The primary clinical justification for screening is the possibility of identifying treatment-eligible infants before irreversible neurological damage occurs and thus enabling timely treatment with arsa-cel for early-onset MLD, or surveillance and possible HSCT for selected later-onset forms.

As for Switzerland, MLD appears to be a plausible candidate for NBS expansion, as the centralized screening infrastructure, established metabolic referral framework, and multiple previous panel expansions provide favorable conditions. However, implementation should not be based on analytical feasibility alone but would require clearly defined confirmatory pathways, expert counseling, particularly for subtype prediction in cases involving VUS, access to arsa-cel, psychosocial support for the families, and preferably a national cost-effectiveness analysis. Given the ultra-rarity of MLD and the very high treatment costs, a structured national pilot or monitored implementation would be the most appropriate next step to generate Switzerland-specific evidence while preserving the opportunity for presymptomatic treatment in affected infants.

## 6. Limitations

This review has several limitations. First, the literature search was restricted to English, which may have led to incomplete capture of the evidence base. Second, the evidence base is rapidly evolving, and recent implementation data may not be available yet in the peer-reviewed form. Third, despite large screening cohorts, the number of confirmed MLD cases remains very small, limiting estimates of sensitivity, treatment eligibility, and long-term outcomes. Fourth, laboratory workflows, including tiered algorithms, sulfatide markers and cut-offs, differed across studies and were heterogenous, limiting direct comparability and generalizability. Finally, cost-effectiveness and implementation findings from other countries may not be directly transferable to Switzerland because financing and laboratory frameworks, and treatment access may differ.

## Figures and Tables

**Table 1:** Method Development and Feasibility Studies for MLD NBS (chronological).

Author (Year)	Study Type	1st Tier Assay	2nd Tier Assay	MLD Samples	Control Samples	Key Findings
Meikle et al. (2006) [36]	ARSA protein quantification methodology	Multiplex immunoassay, 11 lysosomal enzymes incl. ARSA protein in DBS	-	185	599 ^1^	Successful retrospective identification of MLD and ARSA-PD variants.
Barcenas et al. (2014) [46]	1st tier feasibility	LC-MS/MS, 18 sulfatide species in DBS and DUS	-	14	50	Feasibility of sulfatide LC-MS/MS established, significant MLD/non-MLD overlap in DBS, superior separation in DUS.
Spacil et al. (2016) [50]	1st tier improvement	LC-MS/MS, 19 sulfatide species in DBS and DUS	-	19 ^2^	60 ^3^	C16:0-OH and C16:1-OH identified as primary markers, C18:0, C18:1, C20:0 as complementary markers.
Ridsdale et al. (2017) ^4^ [56]	Reinforced need for multi-tiered screening algorithm	-	LC-MS/MS, ARSA activity in DBS ^5^	-	95,163	Supports LC-MS/MS ARSA activity as 2nd tier test. ^6^
Saville et al. (2017) [37]	1st tier improvement	LC-MS/MS, 14 sulfatide species in DBS and plasma	-	19 ^7^	807 ^8^	C18:0 sulfatide is the most discriminating biomarker.
Hong et al. (2020) [32]	2nd tier feasibility	LC-MS/MS, C16:0 in DBS	LC-MS/MS, ARSA activity ^9^	34–78 ^10^	524 ^11^	First antibody-free DBS ARSA activity assay, high sensitivity for trace residual enzyme activity.
Hong et al. (2021) [30]	2nd tier population feasibility	LC-MS/MS, C16:0 in DBS ^12^	LC-MS/MS, ARSA activity ^13^	15 ^14^	27,320	Population-scale feasibility proven: 1 potential MLD case identified, 1 carrier. ^15^
Pettazzoni et al. (2023) ^4^ [55]	1st tier improvement	LC-MS/MS, C16:0, C16:0-OH, C16:1-OH in DBS	-	10	250	Multi-species C16 combination superior to C16:0 alone for first-tier discrimination, confirms feasibility of the multi-species screening approach.
Bekri et al. (2024) [47]	Multi-center study, 1st tier improvement	LC-MS/MS, C16:0 and C16:1-OH in DBS ^16^	LC-MS/MS, ARSA activity ^13^	40	135,784	Combination of C16:0 and C16:1-OH superior to single C16:1-OH or single C16:0 for reducing 1st tier FPR. ^17^
Wu et al. (2024) [49]	UK Pre-pilot; 1st tier improvement, cut-off revision	LC-MS/MS, C16:0 in DBS ^18^	LC-MS/MS, ARSA activity ^13^	20 ^19^	3687	One confirmed MLD case after cut-off revision with a homozygous pathogenic variant associated with LI MLD. ^20^
Sidorina et al. (2026) [59]	1st tier improvement	LC-MS/MS, C16:0, C16:0, C16:0-OH, C16:1-OH, C18:0 DBS and plasma	-	4 ^21^	310 ^22^	C16:0- and C16:1-OH superior in MLD. C16:0 and C18:0 also elevated in other neurometabolic disorders.

^1^ A total of 426 healthy newborn samples and 173 healthy adult samples. ^2^ A total of 14 patients with already diagnosed MLD (11 LI, 3 EJ), 5 newborns who developed MLD (3 LI, 2 EJ). ^3^ A total of 50 non-MLD samples + 10 ARSA-PD samples. ^4^ Conference abstract—full peer-reviewed publication not available at time of systematic review. ^5^ Multiplex immunoassay: 12 lysosomal enzymes, including ARSA protein. Cut-off: 1st percentile of study population. ^6^ A total of 73 (0.08%) samples below 1st-percentile cutoff, gene analysis revealed 51 ARSA-PD, 20 heterozygous, and 2 homozygous. No confirmed MLD cases. ^7^ A total of 18 MLD plasma samples and 1 MLD DBS card. ^8^ A total of 563 healthy controls and 244 controls affected with other storage disorders (Gaucher, Fabry, Krabbe, Tay-Sachs, Niemann–Pick C) and ARSA-PD. ^9^ Assay performed on leukocyte lysate and DBS. ARSA activity cutoff: <20% of daily control mean. ^10^ Leukocyte assay: 22 MLD patients, immunoprecipitation DBS assay: 4 MLD patients, size-exclusion DBS assay: (fresh) 34 MLD patients, (aged) 18 MLD patients. Sample overlap mentioned but not fully disclosed, precise sample size not determinable. ^11^ Leukocyte assay: 1 healthy adult donor, 1 MSD patient; immunoprecipitation DBS assay: 7 healthy adults, 1 MSD patient; size-exclusion DBS assay: (fresh) 10 healthy adults, 3 MSD patients, 294 random newborn samples; (aged) 2 MSD patients, 205 random newborn samples. Possible sample overlaps. ^12^ C16:0 sulfatide cut-off: ≥0.170 μM. ^13^ ARSA enzyme activity cut-off: <20% daily mean. ^14^ A total of 10 LI MLD and 5 EJ MLD. ^15^ First-tier screen positives (false positive rate, FPR), 195 (0.71%); second-tier positives, 2; third-tier positives (ARSA gene sequencing using next-generation sequencing), 1 biallelic (potential MLD case) and 1 heterozygote MLD (carrier) genotype. Confirmation testing could not be completed owing to retrospective de-identified study design. ^16^ Four NBS centers: University of Washington (USA), Manchester (UK), Rouen University Hospital (France), Archimedlife (Austria). Cut-offs: C16:0 ≥ 1.65 multiples of the median (MoM) and C16:1-OH ≥ 2.70 MoM. ^17^ 1st tier C16:0 FPR: 1.8%, 1st tier C16:0-OH FPR: 0.048%, combined FPR: 0.031%. ^18^ Initial cut-off: ≥1.8 MoM, revised to ≥1.65 MoM after identification of a false negative. ^19^ A total of 15 LI MLD samples, 5 EJ MLD samples. Additionally, 2 of 20 would have been missed at the ≥1.8 MoM cut-off, prompting revision. Initial test validation with 6 LI, 5 EJ, 1 LJ, 1AD—total of 13 MLD samples, with 2nd tier testing performed in 12 of these. ^20^ First-tier FPR, 0.30%; 2nd-tier FPR, 0%. ^21^ A total of 1 LI and 3 EJ MLD samples. ^22^ A total of 122 plasma and 188 DBS controls, 85 patients with other neurodegenerative metabolic diseases. ARSA, arylsulfatase A; DBS, dried blood spot; DUS, dried urine spot; EJ, early juvenile; FPR, false positive rate; LC-MS/MS, liquid chromatography-tandem mass spectrometry; LI, late-infantile; MLD, metachromatic leukodystrophy; MoM, multiples of the median; MSD, multiple sulfatase deficiency; NBS, newborn screening.

**Table 2:** Prospective regional and national pilot programs and national implementations of MLD NBS.

Author (Year)	Country	Study Type	1st Tier Screen	2nd Tier Screen	3rd Tier Screen	Sample Size	Outcomes (FPR)	Confirmed MLD	Outcomes
***Prospective Pilot Studies***									
Laugwitz et al. (2024) [34] and Laugwitz et al. (2026) [57] ^1^	Germany/Austria	Prospective pilot September 2021—ongoing	C16:0, C16:1-OH	ARSA activity	NGS: ARSA, SUMF1, PSAP	359,282 (updated)	1st tier: 381 (0.35%) 2nd tier: - 3rd tier: 9 (updated)	9 (updated)	A total of 7 early-onset (4 LI, 3 EJ): arsa-cel initiated; 2 late-onset: HSCT planned.
Malvagia et al. (2025) [48] ^2^	Italy (Tuscany)	Prospective pilot November 2023—ongoing	C16:0, C16:0-OH, C16:1, C16:1-OH	ARSA activity	-	42,262 (target: 80,000)	1st tier: 305 (0.72%) 2nd tier: 10 (0.02%) 3rd tier: -	0	No confirmed prospective cases; retrospective validation successful.
Vasco et al. (2026) [60] ^3^	Italy (Lombardy)	Prospective pilot July 2024—ongoing	C16:0, C16:0-OH, C16:1, C16:1-OH	ARSA activity	WES: ARSA, SUMF1, PSAP	16,130 (target: 100,000)	1st tier: 1305 (6.41%)–247 (1.53%) after retest 2nd tier: 58 (0.36%) 3rd tier: 0	0	No confirmed cases. A total of 33 (0.2%) ARSA-PD cases. Retrospective validation successful.
Kelly et al. (2024) [41] ^4^	USA (New York), “ScreenPlus”	Multi-disorder prospective pilot 2021—ongoing	C16:0	ARSA activity	NGS	>100,000 planed	-	-	No MLD-specific outcomes published to date: program ongoing.
According to Bekri et al. (2024) [47] and Laugwitz et al. (2025) [26] ^5^	France, Saudi Arabia	Assay validation, prospective pilots	Sulfatide quantification	ARSA activity	-	-	-	-	No results published to date: programs presumably initiated.
***National Implementation***									
Norway [25]		Nationwide January 2025—ongoing	Sulfatide quantification	ARSA activity	Genetic confirmation	The first country worldwide to implement nationwide MLD NBS, added in June 2024; the full algorithm is not published.			
USA (New York State) [23]		Statewide pilot September 2025—ongoing	Sulfatide quantification	ARSA activity	ARSA sequencing	First US state to conduct a statewide pilot screening program.			
USA (Federal RUSP) [22,24]		Federal recommendation December 2025	Sulfatide quantification	ARSA activity	ARSA sequencing	MLD added to RUSP on 16 December 2025.			
USA (Pennsylvania) [62]		Statewide implementation January 2026	Sulfatide quantification	ARSA activity	ARSA sequencing	First US state to approve and implement MLD NBS.			
USA (Illinois) [24,64]		Statewide implementation March 2026	Sulfatide quantification	ARSA activity	ARSA sequencing	Second US state to implement MLD NBS.			
USA (Minnesota, Maryland, Utah, Tennessee) [31,36,55,56,57]		Implementation pending	Sulfatide quantification	ARSA activity	ARSA sequencing	Approval has been granted; implementation is pending.			
Switzerland (projected)		Projected national implementation	C16:0 + C16:1-OH (LC-MS/MS)	ARSA activity	ARSA, SUMF1, PSAP sequencing	Formal implementation not yet considered			

^1^ Extended to Austria in 2023. Updated figures per Laugwitz et al. (2026) preprint (September 2021–July 2025) [57]—original NEJM report (Laugwitz et al., 2024) [34] covered 109,259 newborns with 3 confirmed MLD cases. First tier cut-offs, C16:0 ≥ 0.17 μmol/L and C16:1-OH ≥ 0.05 μmol/L; second tier cut-off, <0.015 μmol/L/h. MLD samples cross-validated with Bekri et al. (2024). Of 381 1st-tier positives, only 230 had DBS available for 2nd-tier ARSA activity testing owing to early technical challenges and storage time constraints; all 381 received 3rd-tier NGS. Additionally, 20 had low ARSA activity. ^2^ Initial 1st tier cut-offs at the 99.9th percentile of 5500 healthy newborn samples: C16:0 ≥ 0.196 μM, C16:0-OH ≥ 0.228 μM, C16:1 ≥ 0.041 μM, C16:1-OH ≥ 0.060 μM, sum ≥ 0.485 μM. Lowered to the 99.0th percentile from November 2024: C16:0 ≥ 0.161 μM, C16:0-OH ≥ 0.182 μM, C16:1 ≥ 0.033 μM, C16:1-OH ≥ 0.050 μM, sum ≥ 0.398 μM. Second tier cut-off: <20% daily controls. Successful validation with 23 neonatal and adult MLD DBS. First-tier positives (FPR) before and after cut-off revision: 90 (0.21%) vs. 305 (0.72%). Of 90 1st-tier positives, 10 (0.02%) were recalled for a second DBS sample owing to low ARSA activity (*n* = 6) or insufficient residual material for second-tier testing (n = 4), ARSA activity was normal in all 10 re-tested samples. One additional sample with elevated C16:0-OH and C16:1-OH on repeated testing proceeded to genetic analysis and was classified as a false positive. ^3^ Full-term newborns with birth weight > 2500 g were included; 16,130 of 24,598 (65.6%) eligible newborns were enrolled from July 2024 to July 2025. First tier: C16:0 or a sum of 4 sulfatides ≥97.5th percentile of 5000 newborns (C16:0 ≥ 160 nM, sum ≥ 256 nM) triggered retesting; mean ≥99th percentile (C16:0 ≥ 167 nM, sum ≥ 270 nM) triggered 2nd-tier testing; cut-offs changed in May 2025. Second tier cut-off: ARSA activity <20th percentile of 145 reference newborns. Third tier (WES: ARSA, PSAP, SUMF1): Of 33 newborns with variants, 31 with ARSA-PD and 2 with a heterozygous pathogenic ARSA or SUMF1 variant. ^4^ A total of 14 disorders were screened, including MLD, aiming to generate real-world feasibility and family-impact data. ^5^ France, Hospices Civils de Lyon: assay validation study; France, Rouen University Hospital; and Saudi Arabia, King Fahad Medical City Hospital: prospective pilot studies. ARSA, arylsulfatase A; DBS, dried blood spot; HSCT, hematopoietic stem cell transplantation; LC-MS/MS, liquid chromatography-tandem mass spectrometry; MLD, metachromatic leukodystrophy; MoM, multiples of the median; NBS, newborn screening; NGS, next-generation sequencing; PSAP, prosaposin gene; RUSP, Recommended Uniform Screening Panel; SUMF1, sulfatase modifying factor 1 gene; WES, whole-exome sequencing.

## Data Availability

The data underlying this article are available in the article and can be shared on reasonable request to the corresponding author.

## Supplementary Materials

The following supporting information can be downloaded at https://www.mdpi.com/article/10.3390/ijns12040086/s1, File S1: PRISMA 2020 Checklist; Table S1: Summary of the detailed search strings used for each of the electronic databases, conducted on 29 September 2025; Table S2: Summary of inclusion and exclusion criteria; Table S3: Risk of Bias Assessment; Table S4: GRADE summary of findings: quality of evidence for MLD NBS.

## Abbreviations

The following abbreviations are used in this manuscript:

AD	Adult Disease
AGREE II	The Appraisal of Guidelines for Research and Evaluation II Tool
ALD	Adrenoleukodystrophy
AMSTAR 2	A Measurement Tool to Assess Systematic Reviews
ARSA	Arylsulfatase A
ARSA-PD	Arylsulfatase A Pseudodeficiency
CNS	Central Nervous System
DBS	Dried Blood Spot
DUS	Dried Urine Spot
EJ	Early Juvenile
FPR	False Positive Rate
GRADE	Grading of Recommendations, Assessment, Development, and Evaluation
HSCT	Hematopoietic Stem Cell Transplantation
LI	Late Infantile
LJ	Late Juvenile
LC-MS/MS	Liquid Chromatography—Tandem Mass Spectrometry
MLD	Metachromatic Leukodystrophy
MoM	Multiples of Median
MRI	Magnetic Resonance Imaging
MSD	Multiple Sulfatase Deficiency
NBS	Newborn Screening
NGS	Next Generation Sequencing
NOS	Newcastle–Ottawa Scale
QUADAS-2	Quality Assessment of Diagnostic Accuracy Studies 2
VUS	Variants of Uncertain Significance
WES	Whole-Exome Sequencing
