# Supplementary material for: A Systematic Review of Metachromatic Leukodystrophy Newborn Screening: Transitioning from Pilot Studies to the Real World
Source: Int J Neonatal Screen. 2026 Oct 17;12(4):86. doi: 10.3390/ijns12040086 (PMC13692539; doi:10.3390/ijns12040086)
Supplement: Supplementary file 1 [file IJNS-12-00086-s001.zip › IJNS-4522893-Supplementary Table S1-S4.pdf]

Supplementary material for "A Systematic Review of Metachromatic Leukodystrophy Newborn Screening: Transitioning from Pilot Studies to the Real World" in *International Journal of Neonatal Screening* (IJNS-4522893)

Supplementary Table S1. Summary of the detailed search strings used for each of the electronic databases, conducted on 29th September 2025. Date limitations for updated literature review from 29th September 2025 to 21st September 2026 are added below the respective database search string when applicable.

Electronic database	Search string
Pubmed	((Leukodystrophy, Metachromatic[MeSH Terms]) OR (Metachromatic leukodystrophy) OR (Metachromatic leukodystrophy MLD)) AND ((Neonatal Screening[MeSH Terms]) OR (Neonatal Screening) OR (Newborn Screening))
Ovid - Medline	("Leukodystrophy, Metachromatic"/ or "Metachromatic leukodystrophy".mp. or "Metachromatic leukodystrophy MLD".mp.) and ("Neonatal Screening"/ or "Neonatal Screening".mp. or "Newborn Screening".mp.) [mp=title, book title, abstract, original title, name of substance word, subject heading word, floating sub-heading word, keyword heading word, organism supplementary concept word, protocol supplementary concept word, rare disease supplementary concept word, unique identifier, synonyms, population supplementary concept word, anatomy supplementary concept word]
	limit 1 to dt=20250930-20260921
Embase	('metachromatic leukodystrophy'/exp OR 'metachromatic leukodystrophy' OR 'metachromatic leukodystrophy mld') AND ('newborn screening'/exp OR 'newborn screening' OR 'neonatal screening'/exp OR 'neonatal screening')
	AND [29-09-2025]/sd NOT [22-09-2026]/sd
Scopus	("Metachromatic Leukodystrophy" OR "Metachromatic Leukodystrophy MLD") AND ("Neonatal Screening" OR "Newborn Screening")
	AND ORIG-LOAD-DATE AFT 20250929 AND ORIG-LOAD-DATE BEF 20260921
Web of Science	ALL=(("Metachromatic Leukodystrophy" OR "Metachromatic Leukodystrophy MLD") AND ("Neonatal Screening" OR "Newborn Screening"))

Supplementary Table S2. Summary of inclusion and exclusion criteria.

Category	Inclusion criterion	Exclusion criterion
Topic	Focus on MLD and NBS	Unrelated disorders or not referencing NBS
	Diagnostic algorithms	Treatment focus
Language	English language	Non-English language
Participants	Human studies, newborn population undergoing NBS	Animal or non-human studies, adult data only
Outcomes	Screening methods, feasibility including test accuracy, scalability, confirmatory pathways, patient outcomes, analytical and clinical performance, cost-effective analysis	-

No restrictions to study design, interventions, publication type, publication date, report status, comparators, or country.

Supplementary table S3. Risk of Bias Assessment.

Study	Design	Tool	Risk of Bias
Meikle et al. 2006	Methodology	QUADAS-2	Some concerns
Spacil et al. 2016	Methodology	QUADAS-2	High
Saville et al. 2017	Methodology	QUADAS-2	High
Barcenas et al. 2014	Method/Feasibility	QUADAS-2	High
Hong et al. 2020	Method/Feasibility	QUADAS-2	Some concerns
Hong et al. 2021	Method/Population Feasibility	QUADAS-2	Low
Wu et al. 2024	Pre-pilot, Feasibility	QUADAS-2	Some concerns
Bekri et al. 2024	Multi-center, Method/Feasibility	QUADAS-2	Some concerns
Sidorina et al. 2026	Methodology	QUADAS-2	High
Malvagia et al. 2025	Prospective pilot	QUADAS-2	Low
Laugwitz et al. 2024 (NEJM)	Prospective pilot	NOS	Poor
Vasco et al. 2026	Prospective pilot	QUADAS-2	Some concerns
Laugwitz et al. 2026 (preprint)	Prospective pilot	NOS	Fair (moderate)
Schoenmakers et al. 2025	Descriptive case series	NOS	Poor to Fair (moderate)
Laugwitz et al. 2025	Systematic review	AMSTAR-2	Moderate confidence
Adang et al. 2024	Consensus guideline	AGREE-II	Some concerns
Laugwitz et al. 2024 (EJPN)	Consensus guideline	AGREE-II	Some concerns
Laugwitz et al. 2026 (EJPN)	Consensus guideline	AGREE-II	Some concerns

AGREE II, The Appraisal of Guidelines for Research and Evaluation II Tool; AMSTAR-2, A Measurement Tool to Assess Systematic Reviews; EJPN, European Journal of Paediatric Neurology; NEJM, New England Journal of Medicine; NOS, Newcastle-Ottawa Scale; QUADAS-2, Quality Assessment of Diagnostic Accuracy Studies 2.

Supplementary table S4. GRADE summary of findings: quality of evidence for MLD NBS.

Outcome	No. of studies	Risk of bias	Inconsistency	Indirectness	Imprecision	Publication bias	GRADE rating
**Screening test performance** (screen-positive rate, false positive rate, 1st tier cut-off optimization)	Ridsdale et al. 2017 [1]<br>Hong et al. 2021<br>Bekri et al. 2024 [1]<br>Wu et al. 2024<br>Malvagia et al. 2025<br>Vasco et al. 2026	Serious (heterogeneous study designs, mostly retrospective, variable cut-offs)	Not serious	Not serious	Not serious	Possible (industry involvement: Orchard Therapeutics / ARCHIMEDlife)	**Moderate**
**Sensitivity for detecting affected newborns** (false negative rate)	Meikle et al. 2006<br>Barcenas et al. 2014<br>Spacil et al. 2016<br>Saville et al. 2017<br>Hong et al. 2020<br>Hong et al. 2021<br>Pettazzoni et al. 2023 [1]<br>Bekri et al. 2024<br>Wu et al. 2024<br>Sidorina et al. 2026<br>Vasco et al. 2026	Serious (evidence mostly from archived and de-identified DBS, cut-offs partly derived on confirmed cases)	Not serious	Not serious	Serious (rare disease, small cohort sizes)	Possible (positive results more likely to be published)	**Low**
**Confirmatory pathway** (2nd tier ARSA activity, 3rd tier genetics sequencing, differentiation of ARSA-PD, MSD, PSAP, SUMF1)	Hong et al. 2020<br>Wu et al. 2024<br>Bekri et al. 2024<br>Malvagia et al. 2025<br>Shaff et al. 2025 [2]<br>Vasco et al. 2026<br>Laugwitz et al. 2026 (EJPN)	Not serious	Not serious	Not serious	Serious (limited prognostic validation, subtype prediction unvalidated)	Possible (industry involvement: Orchard Therapeutics / ARCHIMEDlife)	**Moderate**
**Patient outcomes of early treatment** (motor and cognitive function, survival, arsa-cel vs HSCT, symptomatic vs pre-symptomatic treatment)	Groeschel et al. 2023 [1]<br>Wu et al. 2024<br>Laugwitz et al. 2024 (NEJM)<br>Laugwitz et al. 2026 (preprint)	Not serious	Not serious	Not serious	Serious (rare disease, small cohort sizes)	Possible (industry involvement with gene therapy manufacturer as sponsor of key trials) [2]	**Low**
**Patient outcomes of full multi-tiered screening pathway** (multi-tiered screening + treatment, integration into national healthcare systems)	Groeschel et al. 2023 [1]<br>Laugwitz et al. 2026 (preprint)<br>Laugwitz et al. 2026 (EJPN)	Very serious (Case report level evidence, very small case number, incomplete follow-up)	Serious (insufficient cases for comparison across programs)	Serious (scalability to high-volume national programs not yet demonstrated)	Very serious (very few cases, incomplete follow-up)	Possible (industry involvement with gene therapy manufacturer as sponsor of key trials) [2]	**Very Low**

[1] No Risk of bias assessment as no conference papers were assessed, [2] No Risk of bias assessment as the paper describes laboratory reagents, [3] Would not reduce effect: pre-symptomatic treatment may inflate apparent benefit, however, true benefit is heavily supported by evidence and sibling comparisons.

ARSA, arylsulfatase A; DBS, dried blood spot; EJPN, European Journal of Paediatric Neurology; GRADE, Grading of Recommendations Assessment, Development and Evaluation; HSCT, hematopoietic stem cell transplantation; MLD, metachromatic leukodystrophy; MSD, multiple sulfatase deficiency; NBS, newborn screening; NEJM, New England Journal of Medicine; PSAP, prosaposin gene; SUMF1, sulfatase modifying factor 1 gene.
